# Clinical traits impacting human tissue transcriptomes

**DOI:** 10.1016/j.xgen.2022.100245

**Published:** 2023-01-11

**Authors:** Kaur Alasoo

**Affiliations:** 1 Institute of Computer Science, University of Tartu, Tartu, Estonia

## Abstract

In this issue of *Cell Genomics*, Garcia-Perez et al.^1^ report a comprehensive and careful association analysis between gene expression and splicing measured by the GTEx Consortium^2^ in 46 human tissues and 21 demographic and clinical traits.

## Main text

**Figure 1:**
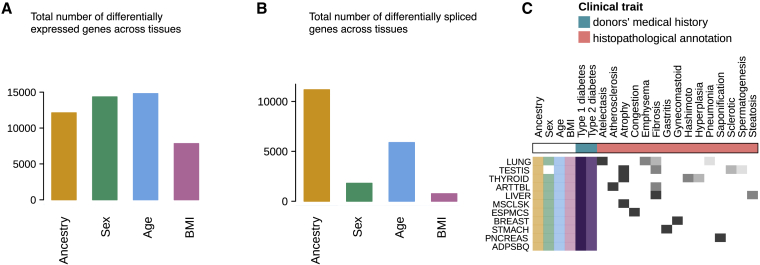
Effects of demographic features and clinical traits on gene expression and splicing levels (A) Number of unique differentially expressed genes associated with each of the four demographic features in at least one tissue. (B) Number of unique differentially spliced genes associated with each demographic feature in at least one tissue. (C) Overview of clinical traits studied and affected tissues. Adapted from Garcia-Perez et al.^1^

In this issue of *Cell Genomics*, the study by Garcia-Perez et al.^1^ expands on previous work in two major ways. While previous studies have typically considered a single tissue^3^^,^^4^^,^^5^ (e.g., blood or brain) or a small number of tissues,^6^ the work by Garcia-Perez et al. investigated 46 different tissues, allowing them to systematically characterize which genes are affected by common demographic features (age, sex, ancestry, body mass index [BMI]) and to determine whether these effects are broadly shared or tissue specific. They found that ancestry, age, sex, and BMI had mostly additive and tissue-specific effects on gene expression, while interactions were rare. Overall, the expression level of most genes was associated with at least one demographic feature in at least one tissue (Figure 1A). Although ancestry had similar effects on splicing to gene expression (both largely explained by *cis*-acting genetic variants), the effects of sex and BMI on splicing were much more limited (Figure 1B), suggesting that largely independent regulatory mechanisms control gene expression levels and splicing. Secondly, the authors expanded their analysis to 17 additional clinical traits extracted from either medical history (diabetes) or donor-matched histopathology images (Figure 1C). The most interesting association was observed between type 1 and type 2 diabetes and gene expression changes in the nerve tissues that correspond to diabetic neuropathy. This association was further validated using histopathology imaging. Together with a previous study focusing on the effect of BMI on gene expression patterns in the adipose tissue,^3^ these studies highlight the value of having matched histopathology images together with tissue gene expression samples.

An important limitation of this study, and many of the previous studies, is that while associations with splicing patterns are easy to detect, they are much more difficult to interpret. This is because reference transcriptome annotations are often incomplete and alternative transcripts or splicing events are strongly correlated with each other. As a result, a high expression estimate for a given transcript does not imply that all exons assigned to that transcript are actually expressed in the sample.^7^ Consequently, the observation that ribosomal genes are often differentially spliced between individuals of European and African American ancestries and that these differences are driven by *cis*-acting genetic variants is an interesting one, but it is unclear whether these changes have any discernable functional impact. Future studies based on long-read RNA sequencing combined with better computational models of protein structure and function will hopefully help to address these issues.^8^

This study neatly highlights the value of combining large transcriptomic datasets with rich phenotyping. However, this is very challenging to achieve at scale, because large biobanks such as the UK Biobank, FinnGen, and other resources focus on recruiting healthy volunteers. This means that they can assemble large collections of phenotypes via questionnaires and linking to registries but are limited by the type of samples that they can obtain from those participants, which is typically frozen whole blood. In contrast, studies like GTEx^2^ have focused on postmortem samples to maximize the number of tissues that can be accessed, but this typically comes at the cost of having access to a much smaller set of organismal phenotypes. Thus, achieving good coverage of both disease phenotypes and tissue samples is likely to remain an ongoing logistical challenge. Most large-scale transcriptomic datasets profiling tissues other than whole blood lack high-resolution phenotyping. A comparable study is perhaps the TwinsUK that has extremely thorough phenotypic information as well as transcriptomic data from four tissues (blood, skin, adipose, and lymphoblastoid cell lines).^3^^,^^6^

The observed association between diabetes and diabetic neuropathy further reaffirms how associations between gene expression levels and disease phenotypes often reflect consequences of disease processes rather than their causes. In a related study, Porcu et al. used a novel design relying on shared genetic associations to link gene expression levels measured in the eQTLGen Consortium^9^ to complex traits measured in completely unrelated cohorts.^10^ This Mendelian randomization approach demonstrated that, in whole blood, most observed correlations between gene expression levels and complex traits are consequences of disease processes rather than their cause. Nevertheless, these associations can yield early biomarkers of the disease, provide important insights about disease progression, and reveal whether and how different tissues are affected by the disease.

Because of its comprehensive design and careful execution, the study by Garcia-Perez et al. is likely to remain an important reference for future disease-transcriptome association studies for many years to come. I expect future single-cell and spatial transcriptomic studies, combined with histopathology imaging, to further clarify the molecular and cellular mechanisms of disease-associated changes.^3^ However, these studies are, at least initially, still likely to focus on easily accessible tissues such as blood. Matching the tissue coverage of GTEx with the phenotypic complexity of large biobanks is likely to remain a major logistical challenge. It is always worth pausing and reflecting on whether the approaches and methodologies that have taken us here are also those that will take us forward. Perhaps there are clever alternatives, like the Mendelian randomization approach proposed by Porcu et al.,^10^ that can achieve similar goals with a smaller logistical footprint. The beauty of science is that we do not have these answers yet.
